# Supplementary material for: MetaQTL: a package of new computational methods for the meta-analysis of QTL mapping experiments
Source: BMC Bioinformatics. 2007 Feb 8;8:49. doi: 10.1186/1471-2105-8-49 (PMC1808479; doi:10.1186/1471-2105-8-49)
Supplement: Additional File 7 — MetaQTL Package : jar file and tutorial. This Zip archive contains both the MetaQTL JAR file and the files of the tutorial. [file 1471-2105-8-49-S7.zip › org.inra.metaqtl/doc/org/metaqtl/bio/util/LGroupPosition.html]

LGroupPosition


|  |  |  |
| --- | --- | --- |
| **Overview** \| **Package** \| **Class** \| **Use** \| **Tree** \| **Deprecated** \| **Index** \| **Help** | |  |
| **PREV CLASS**   **NEXT CLASS** | **FRAMES**    **NO FRAMES**     **All Classes** |  |
| SUMMARY: NESTED \| FIELD \| CONSTR \| METHOD | DETAIL: FIELD \| CONSTR \| METHOD |  |


---


## org.metaqtl.bio.util Class LGroupPosition

```
java.lang.Object
  org.metaqtl.bio.util.LGroupPosition
```

**All Implemented Interfaces:**: ILGroupPosition

**Direct Known Subclasses:**: EstimatedLocusPosition, StaticLocusPosition

---

``` public abstract class LGroupPosition extends java.lang.Object implements ILGroupPosition ```

Class Description Here

**Author:**
:   Jean-Baptiste Veyrieras

---

| **Field Summary** | |
| --- | --- |
| `protected  double` | `absolute` |
| `protected  double` | `stderr` |

**Fields inherited from interface org.metaqtl.bio.util.ILGroupPosition**

`ESTIMATED, STATIC`


**Constructor Summary**

`LGroupPosition(double d)`

`LGroupPosition(double absolute,
double stderr)`


| **Method Summary** | |
| --- | --- |
| `double` | `absolute()` |
| `abstract  int` | `getType()` |
| `static LGroupPosition` | `newEstimatedLocusPosition(double v, double s)` |
| `static LGroupPosition` | `newStaticLocusPosition(double value)` |
| `double` | `standardError()` |

**Methods inherited from class java.lang.Object**

`clone, equals, finalize, getClass, hashCode, notify, notifyAll, toString, wait, wait, wait`

**Field Detail**

### absolute

```
protected double absolute
```

---


### stderr

```
protected double stderr
```


**Constructor Detail**

### LGroupPosition

```
public LGroupPosition(double d)
```

**Parameters:**: `d` -

---


### LGroupPosition

```
public LGroupPosition(double absolute,
                      double stderr)
```


**Method Detail**

### newStaticLocusPosition

```
public static LGroupPosition newStaticLocusPosition(double value)
```

---


### newEstimatedLocusPosition

```
public static LGroupPosition newEstimatedLocusPosition(double v,
                                                       double s)
```

---


### absolute

```
public double absolute()
```

:   **Specified by:**: `absolute` in interface `ILGroupPosition`

---


### standardError

```
public double standardError()
```

:   **Specified by:**: `standardError` in interface `ILGroupPosition`

---


### getType

```
public abstract int getType()
```

:   **Specified by:**: `getType` in interface `ILGroupPosition`


---


|  |  |  |
| --- | --- | --- |
| **Overview** \| **Package** \| **Class** \| **Use** \| **Tree** \| **Deprecated** \| **Index** \| **Help** | |  |
| **PREV CLASS**   **NEXT CLASS** | **FRAMES**    **NO FRAMES**     **All Classes** |  |
| SUMMARY: NESTED \| FIELD \| CONSTR \| METHOD | DETAIL: FIELD \| CONSTR \| METHOD |  |


---
